# Preclinical and clinical aspects of P2X receptors as a common route in different diseases: A meeting report

**DOI:** 10.1007/s11302-024-09994-x

**Published:** 2024-02-20

**Authors:** Luke Tattersall, Elena Adinolfi, Luca Antonioli, Ankita Agrawal

**Affiliations:** 1 The Mellanby Centre for Musculoskeletal Research, Division of Clinical Medicine, School of Medicine & Population Health, The University of Sheffield, Sheffield, UK; 2 Department of Medical Sciences, Section of Experimental Medicine, University of Ferrara, Ferrara, Italy; 3 Department of Clinical and Experimental Medicine, University of Pisa, Pisa, Italy; 4 Department of Clinical Biochemistry, Copenhagen University Hospital Rigshospitalet, Glostrup, Denmark

**Keywords:** P2X, ATP, Purinergic signalling, PRESTO, COST Action

## Abstract

PRESTO was established in 2022 and is a concerted effort by leading European experts in the field of P2XRs and extracellular ATP to promote and advance the transition to the clinic of P2XR-targeting therapies. Following the inaugural meeting in Ferrara which set the foundations of the action and generated interest from many groups and institutes, the second meeting covered the preclinical and clinical aspects of P2XRs as a common route in different diseases, recognising the multidisciplinary and collaborative approach required for a number of medical conditions.

## Introduction

Professor Luca Antonioli and the University of Pisa hosted PRESTO’s COST Action training school and second conference from the 4^th^ to the 7^th^ of September 2023. These PRESTO activities were held in the suggestive location of the Monastero Benedettine on Lungarno Pisa and collected scientists and students from more than 20 Countries inside and outside Europe. The conference was lively, with more than 70 participants who animated constructive discussions, proposed new ideas and started fruitful collaborations.

## The PRESTO COST Action training school: P2X receptors (P2XRs) from basic research to market translation

The first day of the training school intended to give a general overview of P2XR characteristics and pathophysiological roles and a second day focussing on training of the attendees on legislative aspects of patenting and regulations for clinical trials. The school was opened by a lecture from Professor Diego Dal Ben from the University of Camerino, Italy, covering P2XR structure and ion channel properties. The following lectures were from Professor Pablo Pelegrin, addressing the function of P2XRs in inflammation, and Professor Elena Adinolfi, discussing the role of P2XRs in oncogenesis. Finally, Professor Annette Nicke gave an overview of animal models covering P2X null and overexpressing mice and reported on the problems researchers experienced with these animals. The session dedicated to market translation included a lecture by Dr. Alessandra Bosia from Studio Torta, Milan, Italy, a legal expert in patenting. She gave the attendees and other trainers tips to preserve intellectual property rights and described the EU patenting procedures. Finally, Dr. Ileana Frau from IQVIA described procedures and EU legislation to start clinical trials with new compounds and drugs. The training school was enriched by an open, fruitful discussion of each covered subject by both trainers and early career trainees.

## Plenary speakers

The two plenary lectures were given by Professor Francesco Di Virgilio and Professor George Hasko. The lecture by Professor Di Virgilio from the University of Ferrara, Italy covered the latest findings on P2X7R-mediated vesicular release and mitochondrial activity, spanning from cancer to immune cells, including microglia. He described the complex role of the P2X7R, a receptor involved in ATP production and secretion that could also triggered itself by extracellular ATP. Professor Hasko from Columbia University, USA, illustrated his research about the levels of extracellular purines, their receptors, metabolic enzymes and cellular transporters in leukocytes of septic patients. In particular, he showed that since CD39 degrades ATP to AMP, the lower ATP levels in septic individuals may be the result of increased CD39 expression. This increased degradation of ATP did not lead to increased adenosine levels, which may be explained by the decreased expression of CD73, which converts AMP to adenosine. Thus, Professor Hasko demonstrated a differential regulation of components of the purinergic system in PBMCs during human sepsis. The lecture also provided a promising premise to target P2X4R and P2X7R together to reverse macrophage impairment in pathologies involving immune paralysis, organ injury and septic death.

## Preclinical aspects of targeting P2XRs

Preclinical research is used to model different diseases before a novel therapeutic can reach clinical use [1]. This process involves the use of both in vitro and in vivo techniques to determine the possible benefit. The majority of studies presented in Pisa demonstrated in vitro studies, with some presenting further in vivo work. In addition to this, some studies did not model a disease specifically but rather developed further innovative applications. This includes improving agonists/antagonists by Murat Güney through the synthesis of aminopyridine derivatives, improving drug delivery using in silico analysis for eye drop formulations by Todor Dudev, developing P2X-based fluorescent and bioluminescent reporters as tools for tracking the gating of P2XRs and its applications for high-throughput drug screening by François Rassendren or by developing P2X7R PET tracers for imaging of neuroinflammation by Enza Lacivita.

Studies using cell lines and primary cells for preclinical research involved a number of different types including both human and mouse. Immune cells included THP-1, U937, differentiated macrophages and primary human and murine mononuclear phagocytes by Katrin Richter and Veronika Grau for the study of interleukin-1β release and Gloria Lopez-Castejon to study the mitochondrial Pyruvate Dehydrogenase complex. Emel Baloglu used primary rat alveolar type II cells co-cultured with rat alveolar macrophages to demonstrate that inflammation affected alveolar Na-transport. For oncology studies Silvana Morello used human melanoma cells and Ning Wang used the human PCa prostate cancer cell line. Studies into neuronal diseases included the use of pituitary and hypothalamic neurons by Hana Zemkova to test different neurosteroids, cultured neurons by Shai Berlin to examine NMDA receptor function and Valentina Vultaggio Poma used the N13 microglia cell line to study the role of P2X7R in microparticles and mitochondria trafficking in mouse microglia. Other studies included Mehmet Uğur using mice atrial cardiomyocytes and HL-1 cells to show the possible presence of P2X7R and Lin Hua Jiang showing ATP-induced calcium signalling and regulation of mesenchymal stem cells that were derived from human dental pulp tissue.

Animal models are a vital step in preclinical drug development and research [2]. Different models exist for different disease conditions and will differ in species and strain, genetic manipulation, developmental stage and clinical relevance. The presentations in Pisa demonstrated a variety of animal models for studying P2XRs. Three presentations included the use of an experimental autoimmune encephalomyelitis (EAE) mouse model of multiple sclerosis. The studies looked at brain wide P2X7R expression by Andjela Stekic, the expression level of P2X4R and P2X7R in the spinal cord of mice with EAE by Airi Rump and by Carlos Matute who examined the same receptors in demyelination and remyelination with both an EAE and a cuprizone model. In two studies, both Chiara Bianca Maria Platania and Natalia Martínez-Gil used animal models of retinal degeneration to study the role of P2XRs. A single study had an oncology basis, specifically researching prostate cancer bone metastasis by Ning Wang who used an intracardiac injected PCa P2X4R KO cell line into a BALB/c nude mice model.

P2X7R KO mice were used in three studies, one used adult C57BL/6J WT and KO to study the effect of receptor loss in depression by Pál Tod. Chiara Rossi tested whether P2X7R activation might interfere with systemic and cerebral metabolic homeostasis in WT and P2X7R KO mice with a high fat diet. Tobias Engel generated mice lacking exon 2 of the P2X7R gene in either microglia or neurons to show different cell type specific targeting of P2X7R for seizure control.

Other neuronal studies included the use of transgenic APP/PS1-21 and APP23 mice to study ATP metabolism and signalling in microglia for Alzheimer’s disease by Gennady Yegutkin, and Terezia Kiskova used an unpredictable chronic mild stress-induced depression model in Sprague-Dawley rats to test the levels of different P2XRs in the brain following the chronic administration of selected lichen metabolites. Finally, Claudia Verderio showed extracellular vesicles carrying the misfolded protein Aβ contribute to progression of cognitive deficit in the mouse brain.

The studies presented in Pisa showcase a diverse range of mouse models that highlight the versatility and application to perform P2XR related research and make advancements towards clinical use and patient benefit.

## Clinical aspects of targeting P2XRs

P2XRs are relevant to various physiological and pathological processes within the human body [3], various clinical aspects will improve our understanding of their expression, function and role in health and disease and are required before P2XR targeting can be routinely used in humans. This can include biological material, population studies and clinical trials. From the talks in Pisa a number of studies included the use of bodily fluids, including to isolate EVs for stratification of patients in multiple sclerosis by Claudia Verderio, or by using blood samples to detect P2X7R and P2X4R in multiple sclerosis by Airi Rump, blood samples were also used to correlate disease progression in SARS-CoV-2 infection by Anna Lisa Giuliani and to detect P2X7R–induced NLRP3 inflammasome activation by Veronica Canovas as a biomarker in sepsis.

A second important aspect is the use of tissue from patients with different pathologies to correlate expression with disease characteristics. Natalia Martínez-Gil showed P2XR upregulation contributes towards progression in retinal degradation and three studies showed the importance of tissue from epileptic patients; Jens Mikkelsen to determine the binding properties of the JNJ-64413739 to P2X7R to measure neuroinflammation, Tobias Engel who showed human P2X7R expression is elevated in the hippocampus of patients with temporal lobe epilepsy in excitatory and inhibitory neurons, and by Ana Sebastião who examined the role of adenosine in seizures using human epileptic hippocampal tissue, finally Cláudia Valente also used hippocampal slides to examine NLRP3 in Alzheimer’s disease.

Following on from these, two further studies showed the importance of clinical aspects of P2XRs in oncology; Ning Wang demonstrated that the C-terminal truncated P2X7RB isoform but not the wild-type full-length P2X7RA, was positively associated with prostate cancer progression and bone metastases using clinical samples, and Silvana Morello showed using patient serum that patients with melanoma have high levels of extracellular CD73 compared with healthy subjects which was associated with no response to immunotherapy and reduced overall survival and progression-free survival.

Population studies are important to determine genetic differences in P2XRs such as from the occurrence of SNPs. Two studies investigated P2X7R SNPs. Freidrich Koch-Nolte described how the common P2X7R ‘wildtype’ sequence differs from the ancestral allele at three common SNP positions using data from the 1000 Genome Project from ~ 500 people from each of the five major continental regions. Maria Serbanescu used samples from four continents to show that non-synonymous SNPs in P2X7R related to major mood disorders such as bipolar disorder and unipolar major depression.

Finally, two talks discussed factors important to a clinical setting, one talk given by Kris Sachsenmeier from Takeda Pharmaceuticals reviewed why adenosine generation and signalling in cancer has promising results in a preclinical setting but has mixed translatable benefit into human use. The second talk by Jasmina Trojachanec discussed first-in-human trial design considerations, including starting dose selection, study size and population, dose escalation scheme and implementation of adaptive designs according to the recent revision of the European Medicines Agency guidelines on first-in-human trials, to promote safety and mitigate risk. Both of these talks are important to highlight key aspects and challenges associated with P2XR targeting in a clinical setting.

## P2XRs relevance as a common route in different diseases

In addition to the plenary lectures, P2XR-mediated vesicular release, mitochondrial and microglial activation were also the subject of various other talks presented at the conference, including those of Claudia Verderio, Anna Lisa Giuliani, Gennady Yegutkin, Valentina Vultaggio Poma, Gloria Lopez-Castejon and Thomas Duret. Sepsis and inflammation were the focus of several talks during the meeting, including those of Katrin Richter, Veronica Canovas, Kris Sachsenmeier and Cláudia Alexandra dos Santos Valente de Castro. The conference also included talks based on neuronal and muscular diseases, such as those on multiple sclerosis by Carlos Matute and Airi Rump, and those on seizures by Tobias Engel and Ana Sebastiao. A good selection of presentations, such as those of Jens Mikelsen, Enza Lacivita, Hana Zemkova and Terezia Kiskova, was also based on developing new ligands for P2XRs. Friedrich Koch-Nolte, Maria Serbanescu and Pál Tod gave new insights into P2X7R polymorphisms and depression. Silvana Morello and Ning Wang presented studies in oncology.

## Conclusion

Following the success of the inaugural meeting in Ferrara [4]. This meeting was a second opportunity for PRESTO members to meet, interact and highlight some of the key preclinical and clinical aspects of P2XRs as a common route in different diseases from across labs in Europe, whilst also supporting early career researchers with the first training school. The third PRESTO CA21130 COST Action meeting will be held between the 22^nd^ and 24^th^ March at The University of Murcia, Spain, on the topic of eATP and P2XRs in inflammatory conditions, hosted by Professor Pablo Pelegrin and Dr. Juan J. Martinez Garcia. This will be followed by the second training school on the 1^st^ and 2^nd^ of July at The University of Coimbra, Portugal, on the topic of using a bioinformatics approach to P2XR studies, hosted by Dr. Joel P. Arrais. The fourth action meeting will be held on the 3^rd^ and 4^th^ September at the University of Ferrara, Italy, on the topic of P2XRs in diagnostics and medicine, hosted by Professor Francesco Di Virgilio and Professor Elena Adinolfi. This will be followed by the 2^nd^ European Purine meeting which will continue the PRESTO meeting between the 4^th^ and 6^th^ September in Ferrara.
